# A Case of Rocuronium-Induced, Adrenaline-Refractory Anaphylaxis Successfully Treated With Sugammadex

**DOI:** 10.7759/cureus.97832

**Published:** 2025-11-26

**Authors:** Taisei Kawai, Taku Mayahara, Norihiro Shimada, Ryosuke Fukuoka, Yuya Hirai, Masao Uchihashi, Kazuyoshi Matsuura, Tomohiro Katayama

**Affiliations:** 1 Department of Orthopedics, Kobe Ekisaikai Hospital, Kobe, JPN; 2 Department of Emergency and General Medicine, Kobe Ekisaikai Hospital, Kobe, JPN; 3 Department of Anesthesiology, Yukioka Hospital, Osaka, JPN; 4 Department of Anesthesiology, Kobe Ekisaikai Hospital, Kobe, JPN

**Keywords:** adrenaline-refractory shock, anaphylaxis, perioperative allergy, rocuronium, sugammadex

## Abstract

Anaphylaxis during general anesthesia is a rare but life-threatening event, and rocuronium is a leading perioperative trigger. Sugammadex reverses rocuronium-induced neuromuscular blockade by encapsulating free rocuronium molecules. Although sugammadex has been proposed as a potential intervention for rocuronium-induced anaphylaxis, its clinical effectiveness has not been clearly demonstrated. We report an 80-year-old woman who developed severe, adrenaline-refractory anaphylaxis immediately after anesthetic induction for total knee arthroplasty (TKA). Despite fluid resuscitation and repeated doses of adrenaline totaling 3 mg, profound hypotension persisted, and transient pulselessness occurred, requiring brief chest compressions. Sugammadex 400 mg was administered 25 minutes after the onset, while fluid resuscitation and norepinephrine infusion were continued. Within five minutes, the systolic blood pressure improved, and no further adrenaline was required. Subsequent skin testing identified rocuronium as the sole culprit. While experimental and clinical data suggest that sugammadex may not reverse an established immunologic cascade, reducing circulating rocuronium might theoretically limit further antigenic stimulation. Given the dismal prognosis of adrenaline-refractory anaphylaxis, sugammadex may represent a rational adjunctive measure after completion of guideline-directed resuscitation in cases where rocuronium-induced anaphylaxis is strongly suspected. This case underscores both the potential role and ongoing uncertainty surrounding sugammadex in cases of rocuronium-induced anaphylaxis and highlights the need for cautious application and further accumulation of clinical evidence.

## Introduction

Anaphylaxis under general anesthesia is a rare but life-threatening event, with an estimated incidence of about one in 5,000 anesthetics in Japan. Rocuronium, a non-depolarizing neuromuscular blocker, is the most frequently implicated agent [1]. Sugammadex, a selective relaxant binding agent, reverses rocuronium-induced neuromuscular blockade by encapsulating free rocuronium molecules [2]. This theoretical removal of circulating antigen led to its proposal as a potential treatment for rocuronium-induced anaphylaxis. Although multiple case reports have suggested its potential to mitigate rocuronium-induced anaphylaxis [3,4], international guidelines and expert opinions do not currently recommend its use, owing to insufficient evidence and concerns regarding its own potential to cause anaphylaxis [5-7]. We present a case of severe, adrenaline-refractory rocuronium-induced anaphylaxis during anesthesia induction, in which hemodynamic stabilization occurred shortly after sugammadex administration.

## Case presentation

An 80-year-old woman (145 cm, 50 kg) with a history of hypertension, bilateral knee osteoarthritis, and hearing impairment was scheduled for right total knee arthroplasty (TKA). She had previously undergone general anesthesia for small bowel obstruction, ventral hernia repair, and left TKA, all with uneventful rocuronium use. She was not taking β-blockers.

**Figure 1:**
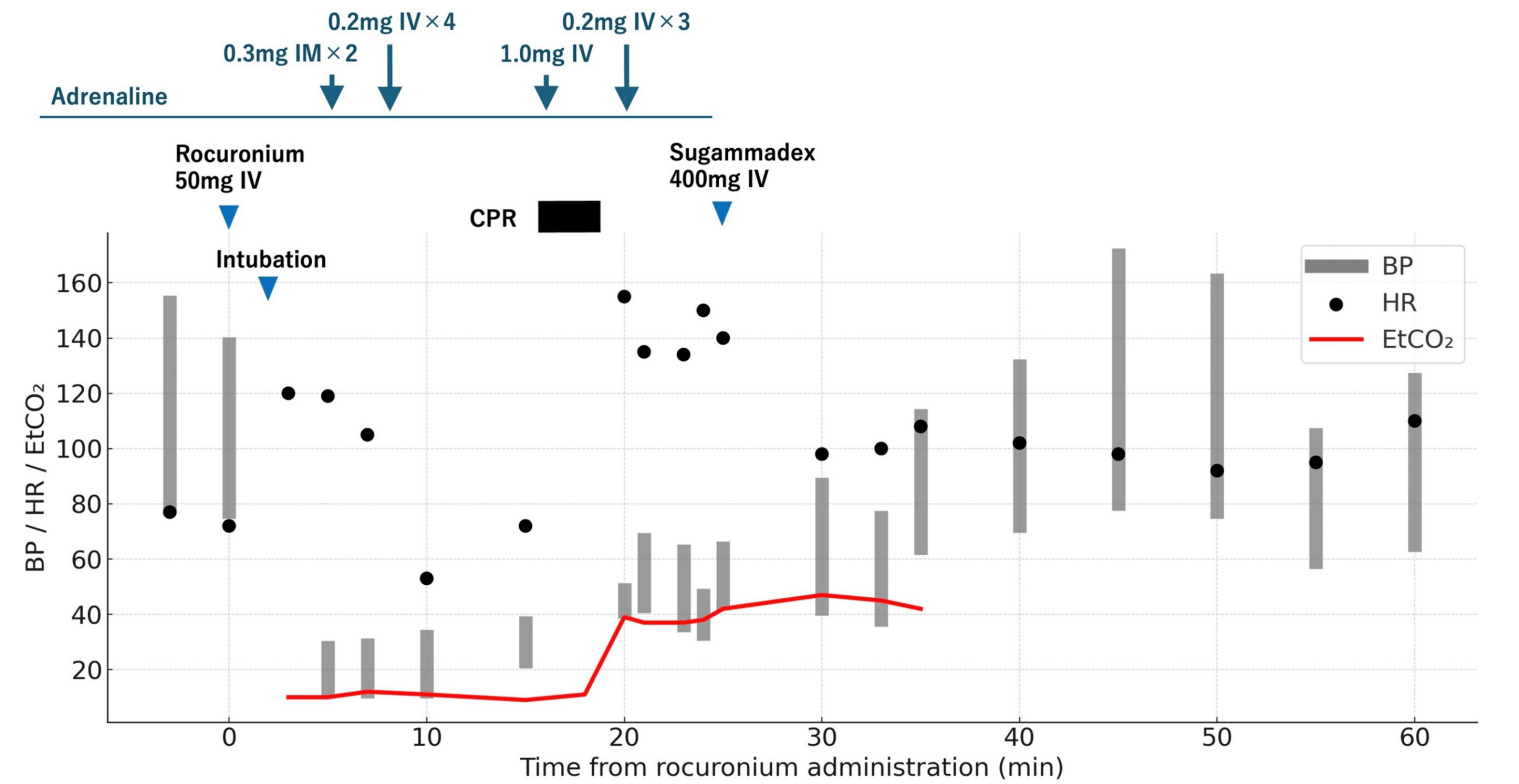
Timeline of anesthetic management and hemodynamic changes. Summary of perioperative events, including administration of rocuronium, adrenaline, and sugammadex, as well as blood pressure (BP), heart rate (HR), and end-tidal carbon dioxide (EtCO₂) trends. CPR: cardiopulmonary resuscitation

Upon arrival in the operating room, her baseline vital signs were a blood pressure of 153/80 mmHg and a heart rate of 77 bpm. Anesthesia induction with intravenous (IV) propofol 50 mg, fentanyl 100 μg, remifentanil 0.1 µg/kg/min, and rocuronium 50 mg was followed by uneventful endotracheal intubation. Immediately after intubation, capnography showed an end-tidal carbon dioxide (EtCO₂) of 10 torr, without any signs of ventilatory difficulty or increased airway pressures. The patient developed profound hypotension (non-invasive blood pressure 28/12 mmHg), an increase in heart rate (120 bpm), and generalized flushing without urticaria, highly suggestive of anaphylaxis. A palpable carotid pulse was still present at that time. Initial management included several IV boluses of phenylephrine, which produced no hemodynamic response, followed by intramuscular (IM) adrenaline 0.3 mg twice and four IV boluses of adrenaline 0.2 mg. A continuous norepinephrine infusion at 0.1 µg/kg/min was also initiated. Despite these interventions, hypotension persisted. Approximately 15 minutes after rocuronium injection, pulselessness was observed, prompting immediate chest compressions. During resuscitation, 1 mg of adrenaline was administered intravenously. Return of spontaneous circulation was achieved after approximately four minutes of cardiopulmonary resuscitation (CPR). This was accompanied by a rise in EtCO₂ to 39 torr, a blood pressure of 49/41 mmHg, and a heart rate of 158 bpm. Bedside transthoracic echocardiography showed no evidence of right ventricular dilation or dysfunction, effectively ruling out massive pulmonary embolism. The patient's systolic blood pressure remained low, ranging from 40 to 70 mmHg, necessitating three additional IV adrenaline 0.2 mg boluses. Hydrocortisone 200 mg, chlorpheniramine 5 mg, and famotidine 20 mg were subsequently given intravenously. Anaphylaxis was strongly suspected, with rocuronium considered the most likely trigger, as no antibiotics had been given. Despite the controversial role of sugammadex in rocuronium anaphylaxis, the decision was made to administer sugammadex, given the refractory nature of shock and the low anticipated risk of harm. Sugammadex 400 mg was administered 25 minutes after rocuronium injection. Within five minutes, systolic blood pressure gradually recovered to 60-80 mmHg, heart rate decreased to 98 bpm, and EtCO₂ subsequently increased to 47 torr. No further adrenaline was required thereafter. In total, 3 mg of adrenaline and 1500 mL of IV fluids were administered in the operating room. Forty minutes after onset, the patient was transferred to the intensive care unit (ICU). On ICU admission, her blood pressure was 170/80 mmHg, and her heart rate was 98 bpm. Norepinephrine was weaned off within 30 minutes. Given the severity of the initial presentation of the anaphylaxis, including transient pulselessness, a low-dose adrenaline infusion (0.02 µg/kg/min) was started in the ICU and continued overnight as a precaution against recurrent hypotension. She was successfully extubated four hours later without any neurological sequelae. The overall perioperative hemodynamic course and treatment timeline are summarized in Figure 1.

Five weeks after the event, skin testing identified rocuronium as the sole causative agent. The postponed TKA was subsequently performed under general anesthesia without neuromuscular blockade, with an uneventful perioperative course.

## Discussion

Numerous individual case reports have described the apparent effectiveness of sugammadex in treating rocuronium-induced anaphylaxis. For example, Takise et al. reported a case where sugammadex was administered six minutes after rocuronium injection, resulting in rapid recovery without the need for adrenaline [3]. Similarly, McDonnell et al. described a patient who suffered cardiac arrest following rocuronium administration and failed to respond to large doses of adrenaline and fluids. In that case, sugammadex was given 19 minutes after rocuronium, and within 45 seconds, spontaneous circulation and blood pressure were restored [4].

However, when data from larger series are examined, the results appear far less consistent. In a retrospective review from Western Australia, eight patients who developed rocuronium-induced anaphylaxis received sugammadex. Transient improvement in blood pressure immediately after administration was observed in three patients; however, none showed a clear clinical benefit attributable to sugammadex [8]. Similarly, a retrospective study from the United Kingdom reviewed seven cases of rocuronium-induced anaphylaxis in which sugammadex was administered. While four patients no longer required vasopressor support after sugammadex administration, the remaining three continued to need vasopressors despite sugammadex use [9].

Experimental evidence provided by Clarke et al. further challenges the hypothesis that sugammadex can reverse established anaphylaxis [10]. In their study, intradermal testing in 10 patients with confirmed rocuronium-induced anaphylaxis showed that rocuronium alone induced localized allergic reactions, and that injecting sugammadex two minutes later failed to suppress them. In contrast, a pre-mixed solution of rocuronium and sugammadex produced no reaction. Based on these findings, the authors concluded that once an anaphylactic cascade has begun, subsequent administration of sugammadex is unlikely to reverse the immunologic response. However, these data reflect localized cutaneous responses in an intradermal model and do not directly translate to the physiology of systemic anaphylaxis.

Additionally, several concerns have been raised regarding the administration of sugammadex for rocuronium-induced anaphylaxis. First, in many clinical situations, the causative agent of anaphylaxis is not immediately clear [9]. The use of sugammadex in such settings may be premature, especially when other agents such as antibiotics or latex could be involved. Second, sugammadex itself is a known trigger of perioperative anaphylaxis. In Japan, it is among the most frequently implicated agents, alongside rocuronium and antibiotics [1]. Third, reversal of neuromuscular blockade may complicate airway management in some circumstances. Kawano et al. reported a case in which sugammadex was administered during rocuronium-induced anaphylaxis while the airway was being managed with a supraglottic device; the patient developed laryngospasm, and ventilation became difficult [11]. Finally, the use of sugammadex may distract clinicians from the timely administration of adrenaline, which remains the first-line treatment for anaphylaxis. In fact, in a case reported by Takise et al., sugammadex was administered promptly after symptom onset, and the patient recovered, but adrenaline was never given [3].

Given these concerns, as well as the absence of supportive evidence beyond isolated case reports, current guidelines and expert opinions remain skeptical about the use of sugammadex in this context [5-7]. For example, the UK guidelines on perioperative anaphylaxis recommend a stepwise approach: initial IM administration of adrenaline, followed by IV adrenaline in escalating doses, if necessary. When the response remains inadequate, the addition of vasopressors such as norepinephrine, vasopressin, or glucagon is advised. In the event of cardiac arrest, standard resuscitation measures should be initiated, with consideration of extracorporeal life support if feasible [6]. Notably, even in cases where rocuronium is the suspected trigger, the guidelines state that “sugammadex has no immediate role in resuscitation of suspected anaphylaxis" [6]. However, whether this position is fully justified remains uncertain.

The three fundamental pillars of anaphylaxis management are the prompt administration of sufficient doses of adrenaline, aggressive fluid resuscitation, and rapid elimination of the triggering agent [12]. The finding by Clarke et al. that a pre-mixed solution of rocuronium and sugammadex failed to elicit an allergic response should not be overlooked [10]. Although sugammadex may not be able to halt an allergic cascade once it has been initiated, its administration may still have value in removing the causative agent and preventing further antigenic stimulation. From this perspective, the potential role of sugammadex in refractory rocuronium-induced anaphylaxis cannot be entirely dismissed.

Adrenaline-refractory anaphylaxis represents an extremely critical and life-threatening situation. In a large European registry including 11,596 cases of anaphylaxis, cardiac arrest occurred in 43% of patients whose anaphylactic shock did not respond to two doses of adrenaline, and the mortality rate reached 26% [13]. In the present case, despite repeated doses of adrenaline totaling 3 mg, including during a transient cardiac arrest, the patient remained in profound and prolonged shock. According to current guidelines, further escalation of adrenaline, consideration of vasopressin or glucagon, and the initiation of extracorporeal life support in the event of recurrent cardiac arrest would be appropriate [6]. However, before proceeding to such advanced measures, administration of sugammadex may be considered on a case-by-case basis, particularly when rocuronium-induced anaphylaxis is strongly suspected.

It remains uncertain whether sugammadex was truly effective in this case, and a similar recovery might have occurred even without its administration. Alternative explanations for the improvement, such as spontaneous resolution of mediator release, cumulative effects of prior adrenaline, or redistribution of intravascular volume, are also plausible. These uncertainties are unlikely to be resolved conclusively, as the rarity and unpredictability of severe perioperative anaphylaxis make large-scale randomized trials practically impossible. Even so, when severe anaphylaxis remains refractory despite adequate doses of adrenaline and fluids administered according to established guidelines, sugammadex may still be considered on a case-by-case basis as a rational adjunctive option in cases of suspected rocuronium-induced anaphylaxis.

## Conclusions

This case highlights the potential role of sugammadex as an adjunctive therapy in severe, adrenaline-refractory rocuronium-induced anaphylaxis. Although causality cannot be definitively established, its administration coincided with hemodynamic recovery, underscoring the need for further clinical evaluation and cautious use. Continued accumulation of similar cases is essential to clarify the clinical utility and safety of sugammadex in this setting.
